# Protocol for academic-community collaboration in the Sweet Dreams study, a community-integrated study of time tradeoffs, sleep, and cardiometabolic health among minoritized women

**DOI:** 10.1093/sleepadvances/zpag055

**Published:** 2026-06-23

**Authors:** Kelly G Baron, Sara E Simonsen, Yeny B Arones, Bobbie Bonilla Bermudez, Princess Bombyck, Dorina Lee, Clarisa Medina-Poeliniz, Kiese Mpongo, Valentine Mukundente, Robin Repta, Elsie Star Prescott-Langi, Sharon Talboys, Fahina Tavake-Pasi, Kamaile Tripp-Harris, Jeanette Villalta, Cathy Wolfsfeld, Ivette A López

**Affiliations:** Division of Public Health, Department of Family Medicine and Public Health, University of Utah, Salt Lake City, UT, United States; College of Nursing, University of Utah, Salt Lake City, UT, United States; Spencer Fox Eccles School of Medicine, University of Utah, Salt Lake City, UT, United States; Division of Public Health, Department of Family Medicine and Public Health, University of Utah, Salt Lake City, UT, United States; Spencer Fox Eccles School of Medicine, University of Utah, Salt Lake City, UT, United States; Calvary Baptist Church, Salt Lake City, UT, United States; College of Nursing, University of Utah, Salt Lake City, UT, United States; Spencer Fox Eccles School of Medicine, University of Utah, Salt Lake City, UT, United States; Calvary Baptist Church, Salt Lake City, UT, United States; Division of Public Health, Department of Family Medicine and Public Health, University of Utah, Salt Lake City, UT, United States; National Tongan American Society, Murray, UT, United States; Division of Public Health, Department of Family Medicine and Public Health, University of Utah, Salt Lake City, UT, United States; National Tongan American Society, Murray, UT, United States; Spencer Fox Eccles School of Medicine, University of Utah, Salt Lake City, UT, United States; Best of Africa, West Valley City, UT, United States; MJV Diversity, West Valley City, UT, United States; Division of Public Health, Department of Family Medicine and Public Health, University of Utah, Salt Lake City, UT, United States

**Keywords:** sleep, women, community based participatory research, cardiometabolic

## Abstract

**Study Objectives:**

Women from racially and ethnically minoritized communities experience cardiometabolic health disparities. Insufficient sleep and poor sleep quality are common and potentially modifiable factors that impact cardiometabolic health; however, women face unique challenges to sleep. A bi-directional community-integrated approach is used as a foundation to learn more about sleep in under-researched populations of Utah, resulting in a study approach that is both effective and rigorous.

**Methods:**

We present the protocol for community integration and development of focus groups among female community health workers who represent the four communities participating in this study: African Immigrant, Black/African American, Native Hawaiian/Pacific Islander, and Latina. We describe establishing relationships, initiating the project, and preparing and conducting data collection, analysis, and dissemination plans.

**Results:**

We anticipate the results of this project will provide unique perspectives about sleep in the participating communities, based on the experience of community health workers as community members and trusted leaders of their communities.

**Conclusions:**

This project continues an ongoing partnership between community health workers and academic researchers focused on reducing disparities in cardiometabolic disorders. The results of this project will lead to the identification of future research questions, outreach/education efforts, and development of a plan for future interventions among diverse racial and ethnic groups in our community.

**Statement of Significance**

This project describes the ongoing partnership between community health workers and academic researchers focused on reducing disparities in sleep and cardiometabolic disorders. The results of this project will lead to identification of future research questions, outreach/education efforts, and developing a plan for future interventions among diverse racial and ethnic groups in our community.

## Introduction and Rationale

Women from racial and ethnic minorities in the United States experience well-documented health disparities, particularly in cardiometabolic diseases [1], including prevalence of heart disease among 59 per cent of Black/African American women over the age of twenty [2] and double the prevalence of diabetes among Hispanic women compared to non-Hispanic white women [3]. Hispanics and African Americans also suffer a greater burden from and poorer outcomes related to diabetes compared to non-Hispanic White women, including higher risk for life-changing complications such as kidney disease, blindness, and limb loss [4,5]. Therefore, interventions are urgently needed to reduce risk factors, improve disease management and quality of life.

Disparities in sleep, such as insufficient sleep duration, poor sleep quality, and sleep disorders such as obstructive sleep apnea, are well documented and related to multilevel factors including comorbidities, housing, environment, occupational exposures, and policies [6]. Importantly, sleep is a critical and understudied factor that has the potential for reducing cardiometabolic disorder disparities. However, despite extensive research documenting sleep disparities, there is limited understanding of sleep within the context of women’s lives. Sleep is now included in the American Heart Association’s Life’s Essential 8 as one of the key behaviors for cardiovascular health [7] and in the American Diabetes Association recommendations [8]. Women have unique barriers to healthy sleep, including family and caregiving responsibilities, effects of hormonal changes during periods such as the menopausal transition, and higher rates of sleep disorders such as insomnia [9]. Women from minoritized communities face these challenges within the context of discrimination, socioeconomic challenges and safety challenges. Despite emerging evidence about the impact of sleep disparities, there is limited research focused on mechanisms of sleep disparities among women from diverse racial and ethnic communities.

In this paper, we describe the protocol for the Sweet Dreams/Dulces Sueños/Indoto Zigyoshe study (1R01MD018517). In developing this project, the Community Advisory Board (CAB) requested that publications describe our study as “community-integrated” rather than “community-engaged” due to the role of the CHWs in the CAB as co-researchers with equal roles in the project [10,11]. The overall goal of this study is to use a community-integrated mixed methods study to evaluate the relationships between time tradeoffs between sleep and other activities with cardiometabolic health. The study plans a 1-year period to establish a CAB and collect qualitative data to inform an observational study in years 2–4. Then in year 5, the qualitative and quantitative data will be used to develop an intervention to improve sleep and cardiometabolic disease risk in the communities participating in this study. Our study is guided by principles of community-based participatory research [12]. The research methods involve principles from community-based participatory research, community-engaged research and community-integrated research throughout, including building partnerships, establishing equal relationships between community representatives and academic researchers, and collaborating in selecting the topic of research, research methods, and plans to disseminate to the community.

To this end, the Sweet Dreams/Dulces Sueños/Indoto Zigyoshe Study is led by community health workers (CHWs) from four communities in our city: African Immigrant/Refugee, African American, Native Hawaiian/Pacific Islander, and Hispanic/Latina. The goal of this paper is to describe our study protocol for inclusive processes of engaging with a diverse CAB of CHWs representing select minoritized Utah populations to guide this work. For our purposes, community is geographic as well as common interest and identity characteristics that unite populations with shared concerns, attitudes, and interpretations. An integral part of our project is CHWs as partners in our CAB and study processes [12–14]. CHWs are front-line public health workers who are trusted members of their communities and serve as liaisons between health/social services and their communities. Collaborations with CHW are a critical aspect of our study’s design and conduct.

## Materials and methods

### Establishing guiding principles in the research team

Based on the community based participatory research framework, prior to beginning the project, the academic researchers endorsed the following values [15,16]:

(1) Mutual learning and trust between academic researchers and community partners must be established in the team and fostered throughout the study.
(2) The team needs to have shared goals and objectives. The research topic must be of interest to the academic research team and community partners.
(3) CAB members will have equal partnership in determining the project focus and methods. CAB approval of the protocol is required to begin the project, and CAB members will be co-authors of presentations and publications.
(4) The team will prioritize rapid feedback to participants and community partners in the study, including interim reports and summaries.
(5) The team will utilize effective communication channels familiar and accessible to CHWs (e.g. often favoring WhatsApp and texts versus email).
(6) The team will plan for measurable processes and outcomes.
(7) The team will seek opportunities to share our findings outside of traditional dissemination methods.

Methods for building community-integrated processes throughout the project.

**Figure 1:**
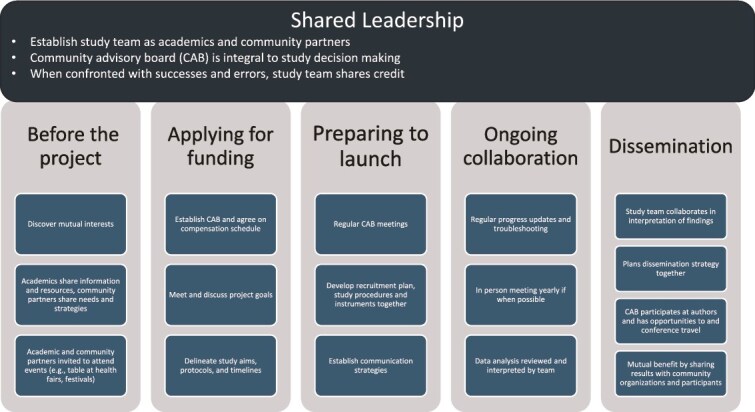
Principals of engagement throughout the project.

In Figure 1, we present our processes for community-integrated framework throughout the project, during each phase of the study.

### Before the project: Developing relationships and selecting a research topic

Community work is much more than asking community-based organizations to partner in a grant, where they agree to be studied [17]. Ideally, building community relationships should predate the study. The community engagement for this project builds on relationships established in a previous study co-led by Dr. Simonsen: the Utah Women and Girl’s Study (UWAG) [18] through the Community Faces of Utah, a collaboration between community leaders, the Utah Department of Health, and the University of Utah. In UWAG, a community needs assessment was conducted to identify the focus and approach of the research [19], and community health workers delivered a culturally tailored wellness coaching intervention for women of color. In UWAG, participants from five racially/ethnically diverse groups worked with CHWs to set goals for healthy lifestyle change. Although this intervention improved diet and physical activity behaviors, results demonstrated that short sleep duration was prevalent but rarely addressed [20]. Upon reviewing these results from UWAG, feedback from the community identified two main overarching needs. First, there is a lack of knowledge about the impact of sleep on cardiometabolic health among both CHWs and community members. Second, participating communities express difficulties and at times pessimism about changing sleep within the context of so many barriers. This was the origin of the study idea, in examining how time for sleep is overshadowed by the many roles and responsibilities of women.

Drs. Baron and Simonsen, academic researchers, then initiated a discussion with CHWs about a Program Announcement by the National Institute of Minority Health and Disparities PAR-20-164 “Mechanisms and Consequences of Sleep Disparities in the US.” Discussions formed the foundation of a study to inform the development of an intervention that incorporates sleep into wellness activities for women in the participating communities. The PIs, Dr. Baron, Simonsen, and Lopez, met with several CHWs who participated in UWAG as well as the Utah Area Health Education Centers’ Coronavirus Aid Relief and Economic Security (CARES) grant. The CARES grant was designed to integrate community health workers in the promotion of COVID-19 vaccination in underserved populations. The discussions were funded by internal research funds allocated to the PI, and CHWs were compensated as research consultants at their hourly rate as CHWs. The academic researchers and CHWs discussed the study opportunity in several brainstorming meetings, first to discuss possible topics and in a final meeting to refine the topic for the grant. The group chose the following topics: (1) the relationship between sleep and blood pressure and diabetes risk in their communities, (2) understanding how busy women make time for sleep in their daily lives, and (3) brainstorming/planning novel interventions that are tailored to the challenges their communities are facing.

### Applying for research funding

The academic researchers selected a funding opportunity and developed a grant proposal based on the discussions with the CHWs. After selecting a topic and proposing an overall structure to the project, the CHWs were asked to submit letters of support for the application. The letters demonstrated their awareness of the topic’s relevance, their previous collaboration with the team, their willingness to participate as a CAB member and in the data collection for the study, their understanding of the planned methods for the study, and compensation for CAB involvement. The budget for the project was developed by the PIs with input from the CHWs to allow for allocation of an hourly rate and number of hours per week and month estimated to complete the study, including recruitment, qualitative interviews, reviewing study results, and data collection. We also budgeted for funds to allow for 2.5 h per CAB meeting, with monthly meetings in years 1 and 5 and quarterly meetings in years 2–4, including one in-person meeting per year with lunch. Finally, we budgeted for an end-of-study dissemination event for CAB members participants, and community members, at a central community location, including refreshments.

### Preparing to launch: establishing a CAB

After a notice of award from NIH was received by the University of Utah (1R01MD018517), the team initiated a CAB for the project. CAB members were drawn, if possible, from the CHW who participated in prior collaborations (UWAG, CARES) and who provided letters of support for the grant application. We recruited at least two CAB members from each of the participating communities (total of 8 community members recruited). CAB members were not required to have any specific training to participate in the CAB and were not selected based on their personal experiences with sleep disorders. Many studies have shown that CHWs have the potential to deliver interventions addressing diverse health disparities around the world [21–23]. However, a few existing studies have collaborated with CHWs in sleep research. When confirming the CHWs for the CAB, we collaboratively established a meeting schedule and compensation schedule that accounted for the time in the meeting and requested work outside meeting time, such as reviewing documents. The expectation of the CAB is that they would provide feedback on all aspects of proposed research, including the appropriateness of research plans, long before starting the study. In addition to the planned CAB meetings, we also scheduled monthly community-specific CAB meetings to discuss any topics that came up that were relevant to their communities. The CAB was encouraged to share events going on in their communities, fundraising, and other events with the research team. CAB meetings were scheduled online with one in-person meeting per year. An essential and often overlooked factor ensuring clear communication with the CAB that we utilized was employing a skilled research coordinator that CAB members could contact with questions (Robin Repta). This research coordinator’s role in managing communication with CHWs who are outside of traditional academia is key in ensuring communications are consistent and delivered in a way that facilitates communication in the desired methods (e.g. not only via email). Further, the study coordinator identifies processes that need modification, streamlining, or clarification. Qualifications for the research coordinator position included a master’s degree or at least 2 years of research experience. The research coordinator for this study has a master’s degree and 15 years of previous research experience as a graduate student and Social Science researcher in Public Health and Gender Studies. Her background included extensive experience conducting community-engaged research with various under-resourced communities in Canada.

### Developing the focus group methods, interview guide and post-focus group survey

The first activity of the project was to complete focus groups with CHWs to assess experiences of time use, sleep, and culture. Focus groups were developed in collaboration with the CAB. Participants in focus groups were women ages 18 and above who had worked or volunteered as a CHW in the past 5 years. The team (researchers and CAB) decided that the focus group facilitators would be CAB members who participate as CHWs in the communities participating in our study. Many of the CAB members already had training and experience as focus group facilitators, but everyone attended a brief training on the basics of facilitation using a semi-structured topic guide in English, Spanish, or Kirundi led by an academic team member with extensive qualitative research experience (Dr. Talboys). Facilitators were trained to use probing questions to obtain more detail about women’s responses and to generate discussion by inquiring about differing views in the group. Focus groups were conducted over Zoom, except for the Latina group, because the CHWs of this group requested an in-person group to accommodate several CHWs who could not connect to the virtual meeting. At least one academic researcher attended each focus group as tech support or note taker. The research team, including CAB members, collaboratively developed the semi-structured interview guide based on research questions and a brief demographic survey for focus group participants. The main domains included: (1) personal beliefs about what is “good” vs “poor sleep,” (2) cultural perceptions of sleep in Utah minority women and their communities, (3) perceived conflicts to sleep duration and quality in their communities and time use priorities, and (4) resilience factors observed that help sleep in their communities. The interview guides were translated to Spanish. Although some study activities were planned to be presented in Kirundi, the African Immigrant CAB members felt that focus groups could be conducted in English for their community. In addition to adapting language, CAB members expressed a desire to adapt the interview guide to their communities, such as the order of questions. The interview guide by community is listed in Table 1. We developed a unique facilitator guide for each focus group, including a PowerPoint presentation with questions and images that were specific to each community. While the overall questions were the same, the icebreaker activities and order of the questions varied by community. At the end of each focus group, the academic researcher presented a brief (10 min) presentation on sleep and strategies to promote sleep health.

**Table 1:** 

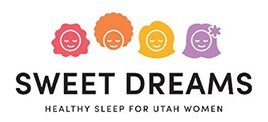
**Focus Group Topic Guide—Latina Version**
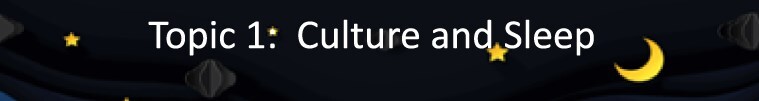
1) ***“Let’s talk about sleep; What are some sleep habits that you think are common in your community, or perhaps in your home country, especially for women?”*** a) *“What is considered a normal sleep routine?” “What times of day or night are common?”* b) *“How are naps parts of your culture?” “What are people’s attitudes about naps?”* c) *“When it comes to sleep, are there any beliefs or practices about sleep that are unique to your culture/community?”*
2) ***“What are some common concerns you hear about sleep? What health issues do people in your community, or family think are related to sleep?”** (Key point – how did parents talk about sleep?)*
3) ***Probes about culturally specific to sleep.*** a) *“If this were you in the picture (see slide), what else would you add to the picture (from a cultural context)?”* b) *“Are there any sayings, beliefs, legends, or myths about sleep that are talked about in your culture?”* c) *“Just for fun, what are some words or phrases people use for sleep in your culture or language (They don’t need to be in English.).”* d) *“In your culture, are there any typical things that affect your sleeping environment, such where people sleep, what kind of bed, or who sleeps in the room?”*
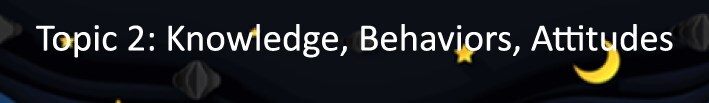
1) ***“Let’s talk about you and your sleep (time use).” “If you think about a 24-h day like pie, what does the sleep piece of the pie look like, including long sleep and naps?”*** a) *“What are some of the other pieces that crowd out sleep?”* b) *“When you are supposed to be sleeping and are not, what are you doing?”* c) *“Does your work compete with your sleep hours? How? In what ways?”* d) *“What other activities interfere with sleep?”* 2) ***“Ideally, how much sleep do you think you need each night and how much do you get?”*** 3) ***“On a scale of 1 – 10, how important is sleep on your health?” “How does it compare to other things, like diet, nutrition, and exercise?”*** 4) ***“What health problems show up or get worse when people have poor sleep?”*** a) *“In your opinion, what does ideal sleep look like?” “What does healthy sleep mean?”* b) *“What are some habits that you think are good for sleep?”* c) *“In your opinion, what does unhealthy sleep look like?” “What habits are not good for sleep?”* d) *“Where have you gotten information about sleep needs?”*
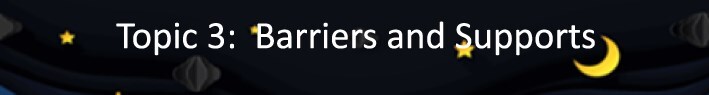
**Barriers:** *Internal,* i.e. *stress, thoughts, and External factors* i.e. *environment, surroundings.* 1) ***“What other things interfere with sleep, worry or tension?” “External things, family, space, environment?” Probes:*** a) *“Sleeping environment, bed/comfort, noise, household, domestic work, kids, screens, phone/TV, weather, temperature etc.).”* b) *“How does your family picture influence your sleep (kids, snoring partner)?”* c) *“Worry or tension?”*
**Supports** 2) ***“When you can’t sleep, or think you are not getting good sleep, what do you do?” Prompts:*** a) *“Chamomile tea, melatonin, night-time mode for phone, glass of wine, etc.”* b) *“Medicine over the counter or Rx?”*
3) ***“At what point would you be worried about your sleep and how it affects your health?”***
4) ***“Have you ever talked to a health provider about sleep issues**?”* a) *“At what point would you consider talking to a provider, what kind?”* b) *“What kind of doctor or health provider would you talk to?”* c) *“How would cost or insurance affect your decision to seek care?”*
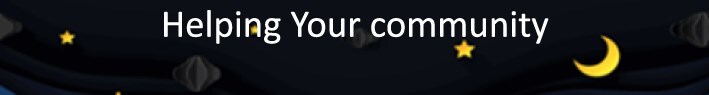
***“As a CHW, what kind of conversations have you had with your community about sleep health? What advice do you give community members?”***
2) ***“As a CHW, do you feel prepared with knowledge and resources to advise people about sleep**?”* a) *“What additional education or training do you need?”* b) *“Why are you ideal to deliver information?”*
3) ***“What suggestions do you have for promoting sleep health in your community?”*** a) *“If you were to have a magic wand, what would you do to help people in your community to develop healthy sleep habits?*” b) *“Think about individuals, families, working conditions, healthcare access, etc.”*
**Wrap up and Move to Education portion (5-10 min).** **Recommendation is to give participants health education materials.**

The team also worked together to create a post-focus group demographic survey. Although this is a seemingly simple task, the CAB met for several meetings to select the questions that would be asked, ensuring that items were culturally sensitive and appropriate. For example, several CAB members felt that asking about income would be uncomfortable, and given the higher prevalence of larger families, it was important to ask about both income and the number of people supported by that income. Survey items included: age, sex, gender, race, ethnicity, birth country, years living in the United States (if relevant), preferred language, marital status, education, household income, number supported by the household income, employment status, shift work, average sleep duration on workdays and non-workdays (or weekdays and weekends), whether they have been diagnosed with sleep apnea, and self-rated health. Participants also completed the brief acculturation scale (4 items) and answered, “In your role as a CHW, how confident are you in talking with other people to promote healthy sleep?”

### Focus group procedures

Focus group participants were all community health workers who were recruited by each focus group leader of the same community. Inclusion criteria included: (1) female (sex at birth and/or gender) and (2) working (either paid or unpaid) as community health worker within the past 5 years. Since there were two focus groups planned for each community, the focus group leader also had the opportunity to participate as a focus group participant if interested. The protocol was approved by the University of Utah Institutional Review Board (IRB_00171102). Focus group participants received a consent cover letter, but written informed consent was not required for this protocol. After the focus group, focus group participants completed a brief demographic survey administered via REDCap. After the focus group, attendees were paid $100 via an online or physical gift card and given a handout on sleep resources developed in collaboration with the CAB, using plain language free from jargon.

### Focus group analysis plan

Focus group analysis is still in progress. We recorded, translated, transcribed, and uploaded transcripts into Dedoose software for coding and analysis (Sociocultural Research Consultants LLC, Los Angeles, CA). Transcripts are being coded using applied thematic analysis [24], which is a set of procedures designed to identify and examine themes from textual data in a way that is transparent, reproducible, and credible. The academic team is conducting thematic analysis in two stages: deductive and inductive. Following Crabtree and Miller’s methods of structured, deductive coding [25,26] the team is reading the transcripts and coding text for three broad domains of the socio-ecological model: individual, interpersonal, and societal domains. In the inductive stage, the team is reviewing the coded text to reach consensus on emerging themes in each domain. The coders are meeting to develop a codebook to define each theme. Coding will be ongoing until we reach thematic saturation. In the last step, the academic team will meet to review the final codes to ensure each is a distinct theme of the discussions. Key quotes will be back-translated to their original language and reviewed by the CAB for accuracy. Demographic surveys will be summarized to provide context about the characteristics of the focus group members.

### Qualitative sample size estimation

We estimated that 2–3 focus groups per community will offer adequate opportunity to capture relevant themes. Prior qualitative health behavior research suggests that thematic saturation tends to occur 90 per cent of the time in 3–6 focus groups [27]. We expect some distinct differences between communities but also overlap. Therefore, we expect 6–8 focus groups will offer adequate opportunity to see themes of interest.

### Ongoing collaboration

The CAB and academic researchers continued to meet regularly throughout the focus group data collection and analysis. Recognizing the importance of balancing in-person relationship building with the strain of travel, we established a yearly face-to-face meeting with lunch and presentations of data analyses in progress.

### Dissemination: delivering feedback

After completion of the first coding, we presented a summary of the main themes and quotes at the annual in-person CAB meeting. Then, after refining the codes, we send to each community a community-specific summary report with main community-specific themes. Our team is also working on the presentations and publications of the focus group data, on which academic partners and CAB members will be co-authors. We plan to submit one overall qualitative paper covering all 4 communities and then a separate paper for each community.

### Next steps after the focus groups and timeline

The goal of the qualitative phase of the study was to inform the development of the next phase, cross-sectional, observational study of sleep, time use, and cardiometabolic health among 400 women (100 women per community). The protocol was designed to include a survey, cardiometabolic assessment, at-home wearable and sleep diary, and a 24-h time use survey. To establish the survey, The CAB met for several meetings to review candidate measures, select proposed measures and review each questionnaire item by item. CAB members answered the surveys and identified items that felt would be inappropriate for a variety of reasons, such as not relevant to their community or too invasive. The themes identified in the qualitative research were compared to the surveys proposed in the grant application, and the survey was adapted to include those areas and remove areas that were less frequently discussed based on focus group findings and CAB input. For example, items were added to assess supplement and sleep aid use and cultural identity/pride. The team also had extensive discussion of appropriate assessment of adverse childhood events. The CAB felt these events were important to assess due to impact on later mental and physical health but requested to use what they felt was culturally appropriate language and established a robust follow-up plan if a participant disclosed a traumatic event and needed a referral for mental health treatment. All items and the order of the survey were approved by the CAB. The CAB tested the cardiometabolic testing protocol, including the stadiometer, automated blood pressure machine, scale and dried blood spot kit. Several different methods were tested in CAB meetings and the team worked to put together a testing kit that could be easily transported to participants’ homes. The CAB identified possible challenges to the cardiometabolic assessment for participants with higher BMI, and recommended that we ensure the team has larger blood pressure cuffs, scales with a higher weight range and larger measuring tape for measuring waist and hip circumference, in order to be inclusive in their populations. The team established an easy to interpret feedback report to provide to participants within 1 month (blood pressure and BMI are available immediately, sleep and glycated hemoglobin available after processing). Several time use instruments were reviewed and tested by the CAB and investigators and a measure was selected for the study. After completion of the quantitative phase, both qualitative and qualitative data will be used in an intervention mapping [28,29] strategy to plan a next step sleep intervention, including reviewing results, identifying theory-based interventions and selecting the intervention components. The final goal of the study is to use this information to generate novel sleep interventions tailored to women from diverse race and ethnic communities, focusing on what are the most critical sleep issues and how to approach sleep interventions in culturally appropriate ways that fit their lives. The study will be conducted over a 5-year period. The focus groups will be conducted in year 1, the field study will be conducted in years 2–4 and the intervention mapping will be conducted in year 5.

## Discussion

### Main findings and what this study adds

The process of developing and sustaining community-integrated research in this study began approximately a decade prior to the current protocol for this particular project. Building on this foundation, this protocol demonstrates our team’s ongoing collaborations in building the multi-phase study of the Sweet Dreams Study/Dulces Sueños/Indoto Zigyoshe, a community-integrated, mixed-methods study of sleep, time use, and cardiometabolic health among women from African Immigrant or Refugee, African American, Native Hawaiian/Pacific Islander, and Hispanic/Latina. Our partnerships with CHWs to establish a CAB and collect qualitative data are a strength of the study, building on their knowledge and skills in community outreach, health promotion, and partnerships with academic researchers. Although the research topic was promoted by the research team based on the existing literature, CHWs provided a blueprint for the areas that surround barriers and facilitators of sleep behaviors. The ongoing process of collaboration, checking with communities, feedback, and capacity building has been critical to the establishment of this process and our continued collaboration with CAB members. The team acknowledges that the CHW’s endorsement and positive regard for the topic and team are critical factors in the success of the study. This ongoing mutual respect is critical due to the history of mistreatment and abuses in research among minoritized communities [30]. Our study also demonstrates a commitment to the sustainability of our research relationships. Regardless of funding, our team has continued to sustain relationships based on common interest and commitment to the public health of all Utahns.

### Limitations

We also acknowledge some potential limitations to our study. As is typical in community-integrated research, developing surveys and interviews is time-consuming and the CAB must be able to slow the process down if they require more time to evaluate the study materials. In our case, we tried to accomplish too much too fast in each of the initial CAB meetings and needed to extend our timeline by several months. We also had changes in CAB membership due to attrition and had to ensure the proper onboarding and training of new members. We also planned an hourly rate for CAB members and did not take into account cost-of-living increases for these consultants, impacting their future earnings for the duration of the study. While cost of living increases are readily made by administrative staff for research faculty, these were not done in consultant agreements (including CHWs). We also need to continue to recognize the opportunity for education and capacity building through this partnership. A salient and recent example includes the development and writing of this manuscript, during which time we learned that CHWs were interested in learning to code qualitative data and being more involved in the data analysis process for the qualitative data obtained in this study. In future studies, we will consider building in training and participation in qualitative analyses for CHWs on our team, if interested. We also acknowledge that the study’s title including “time use” may overemphasize the individual factors in sleep, without considering the contextual and system level barriers. These factors such as work and commute times, school schedules and housing conditions were topics commonly discussed in the focus groups and highly relevant to women’s sleep.

In summary, the process of academic researchers building trust in community-integrated research is more than learning a method of conducting a study, it involves taking the time to show up on the community’s terms, gaining trust and demonstrating flexibility, which is an indispensable part of that trust. Together, we hope to achieve actionable findings, driving home principles of the interdependence of all communities’ health.

## Supplementary Material

SPIRIT_2025_checklist_zpag055
